# OD28 Towards Implementing New Payment Models For The Reimbursement Of High-Cost, Curative Therapies: Insights From Semi-Structured Interviews

**DOI:** 10.1017/S0266462324001594

**Published:** 2025-01-07

**Authors:** Thomas Desmet, Sissel Michelsen, Elena Van den Brande, Walter Van Dyck, Steven Simoens, Isabelle Huys

## Abstract

**Introduction:**

In response to the intricate challenges posed by high-cost, one-shot curative therapies, this study explores what hinders the wide implementation of innovative payment schemes across Europe. Drawing insights from the Belgian social healthcare system, this study focused on defining the necessary and sufficient conditions for implementing outcome-based spread payments in the context of market access to advanced medicinal products

**Methods:**

Semi-structured interviews (n=33) were conducted with physicians (n=2), hospital pharmacists (n=4), hospital managers (n=2), patient representatives (n=3), industry representatives (n=5), Belgian policymakers (n=6), sickness fund representatives (n=4), legislative experts (n=2), and accounting experts (n=5) to elicit opinions and insights on stakeholders’ responsibilities and roles, and identify the necessary and sufficient conditions to establish outcome-based spread payments for the reimbursement of innovative therapies. The interviews took place between July 2020 and October 2020. The framework method analysis was performed using NVivo software (version 20.4.1.851). Statements were allocated into six main topics: payment structure, spread payments, outcome-based agreements, governance, transparency, and regulation.

**Results:**

Interviewees across stakeholder groups endorsed the idea of implementing outcome-based spread payments. However, opinions varied on practical and legal feasibility, especially regarding long-term follow-up for patients, data collection burden on physicians, and implications on the financing flow of health technology developers, hospitals, and the government. Concerns were also raised regarding the potential need for new governance structures, enhanced transparency on agreements and pricing mechanisms, as well as defining data requirements to address uncertainties often seen with this type of therapy. All interviewees emphasized the importance of increasing stakeholders’ understanding of these agreements to foster broader acceptance and successful implementation.

**Conclusions:**

The effective implementation of outcome-based spread payments falls behind because consensus on how this reimbursement method can be a sustainable solution is missing. Leveraging the concepts of necessary and sufficient conditions from organizational research, this study provides guidance on resolving challenges and defines stakeholders’ roles for successfully implementing this reimbursement approach.

